# Outcome of Tetralogy of Fallot Through Initial Palliation and Surgical Repair

**DOI:** 10.1007/s00246-025-04021-1

**Published:** 2025-09-24

**Authors:** Miriam Giacobbe, Trisha V. Vigneswaran, Shuwayne DeSouza, Thomas Witter, Sahar Mansour, Conal Austin, Mathew Jones, Eric Rosenthal, Shakeel A. Qureshi, John M. Simpson, Vita Zidere

**Affiliations:** 1 Department of Congenital Heart Disease, Evelina London Children’s Hospital, London, UK; 2 School of Biomedical Engineering and Imaging Sciences, King’s College London, London, UK; 3 South West Thames Centre for Genomics, St George’s University Hospitals, London, UK; 4 Cardiovascular & Genomics Research Institute, City St George’s University of London, London, UK

**Keywords:** Tetralogy of Fallot, Outcome, Reintervention, Mortality, Congenital heart disease, Cardiac surgery

## Abstract

Tetralogy of Fallot (ToF) is the most common cyanotic congenital heart disease, frequently diagnosed prenatally. To enhance understanding, we reviewed 18 years of our institutional outcomes for patients with classic ToF. There were 410 patients of whom 233/410 (56.8%) were diagnosed prenatally. A genetic abnormality was diagnosed in 38/410 (9.3%), most commonly 22q11.2 microdeletion (43.6%). There were 10/410 (2.4%) deaths in the neonatal period prior to any cardiac intervention. Prior to complete ToF repair, 66/400 (16.5%) of patients required cardiac procedures, in 15 in the first week after birth. Babies with an antenatal diagnosis were more likely to require at least one procedure before complete ToF repair than those with a postnatal diagnosis (*p* = 0.039). There was a trend to shorter median length of stay in patients with RVOT stent (median 5.4 days) or duct stenting (median 4.2 days) versus BTT shunt (median 8 days). Complete ToF repair was undertaken in 396/410 at a median age of 6 months (IQR: 4.0–8.0 months), with a 30-day postoperative survival rate of 99.5%. The actuarial survival for the whole group (*n* = 410) of 410 patients was 98.5% at 30 days, 96.2% at 1 year, and 95.2% at 3 years of age. Following complete surgical repair, reintervention was required in 25% of cases: 3.3% at 30 days, 10.6% at 1 year, and 19.2% at 5 years. The freedom from reintervention was 89.4% after 1 year and 80.8% after 5 years. There were no deaths beyond 2.5 years post-repair. In conclusion, ToF can be repaired with a low procedural mortality rate and promising long-term survival outcomes, but there may be pre-procedural deaths and there is a likelihood of requiring reinterventions during follow-up.

**Supplementary Information:**

The online version contains supplementary material available at 10.1007/s00246-025-04021-1.

## Introduction

Tetralogy of Fallot (ToF) is the commonest form of cyanotic congenital heart disease, occurring in 3–5 per 10,000 live births [1, 2]. Definitive surgical repair of ToF is usually undertaken in the first year of life and has a low risk [3] of mortality, which, over the past several decades, has decreased significantly [4]. The exact timing varies between institutions, with some favoring early neonatal repair [3–5]. In the United Kingdom, the 30-day survival is reported to be 98.2%, from the mandatory, nationally collated procedural database [6].

In some infants with significant cyanosis, an early intervention may be necessary before complete surgical repair. The intervention may be a systemic-to-pulmonary artery shunt, right ventricular outflow tract stent, balloon pulmonary valvuloplasty, or stenting of the arterial duct.

Our study aims to report the short- and medium-term outcomes of ToF, focusing on the need for and the type of intervention before and after complete repair. We describe the genetic associations of ToF and their impact. The length of hospital stay following complete repair in relation to the type of intervention proper to complete repair is also described.

## Methods

This was a retrospective single-center cohort study conducted at the Evelina London Children’s Hospital (United Kingdom), a tertiary congenital heart disease center. All patients with clinical coding of ToF, ToF repair, or Fallot-type repair during the period April 1, 2004, to March 31, 2022 were retrieved from the departmental database – Heartsuite (Heartsuite Lmt) and from our mandatory submissions to the National Congenital Heart Disease Audit (NCHDA) which is part of the National Institute for Cardiovascular Outcomes Research (NICOR) database, which is externally audited for accuracy every year. Data were further collected from our electronic patient record system.

Patients who were operated on at our center but not followed up before and/or after the surgery at our center were excluded from this cohort. For prenatally diagnosed cases, we generally deliver at the cardiac centre if there are features suggesting potential duct-dependency, e.g., reversal of flow in the arterial duct, hypoplasia of pulmonary arteries.

Patients who underwent surgery in the first year of life with key anatomical features of the “classic” tetralogy of Fallot, and patients with “tetralogy type” double outlet right ventricle were also included. However, those with associated atrioventricular septal defect, pulmonary atresia, or absent pulmonary valve syndrome were excluded. The age and weight at the procedure and the type of procedure were recorded. Extracardiac and genetic conditions, when present, were recorded.

The study was registered as a clinical service evaluation with Guy’s & St Thomas’ NHS Trust as per Institutional Policy (registration number: 15416) and ethical approval was not required.

Statistical analyses were performed using R software version 4.3.3 (R Foundation for Statistical Computing, Vienna, Austria). The Shapiro–Wilk normality test was used to evaluate the distribution of continuous variables. For group comparisons of continuous variables, the Kruskal–Wallis rank sum test and Wilcoxon rank sum test were applied to non-parametric data, while Welch’s t-test was used for parametric data. Categorical variables were compared between groups using the Chi-square test. Statistical significance was assessed using two-sided tests, with a significance level set at *p* < 0.05.

Kaplan–Meier (KM) estimates were used to calculate the actuarial survival curve, and the freedom from intervention following ToF repair.

Cox proportional hazards models were used to assess the predictors of postoperative length of stay (LoS), with hospital discharge defined as the event of interest following complete repair of ToF. Univariable models were first fitted for each predictor. Variables with a univariable *p*-value < 0.1 were included in the multivariable Cox proportional hazards model. Results are reported as hazard ratios (HRs) with corresponding 95% confidence intervals (CIs). An HR < 1 indicates a lower hazard of discharge, corresponding to a longer LoS.

## Results

During the eighteen-year period (April 1, 2004, to March 31, 2022), a total of 410 patients who met the inclusion criteria for ToF were identified from the database and included in the analysis. All 410 patients had electronic records, including echocardiography and surgical reports and clinical documentation available for review. Of these, 233/410 (56.8%) were prenatally diagnosed. The patient follow-up period ranges from 0 to 18 years*,* with a median follow-up of 8 years (IQR 4 years to 12 years).

### Mortality Before any Cardiac Intervention

There were 2.4% (10/410) patients who were diagnosed with ToF but died in the neonatal period before any cardiac procedures. Causes of death were recorded as follows: related to trisomy 18 (*n* = 1), severe lung disease (*n* = 6), complications after tracheo-oesophageal fistula repair (*n* = 2), and tracheal stenosis with inability to ventilate (*n* = 1). Six of these ten patients were born before 37 weeks of gestation.

### Genetic Associations and Comorbidities

Pathogenic genetic abnormalities, chromosomal and monogenic disorders, were present in 39/410 patients (9.5%), as shown in Table 1. Extracardiac anomalies including gastrointestinal (*n* = 17), brain (*n* = 12), respiratory/ear, nose, and throat (*n* = 15), renal (*n* = 10), and skeletal (*n* = 6) were found in 14.6% (60/410) of patients of whom 10 had confirmed co-existent genetic abnormalities. There were 43 patients born < 37 weeks’ gestational age, including 11 patients with a genetic abnormality.

**Table 1:** Pathogenic genetic abnormalities: chromosomal and monogenic disorders diagnosed in the study group

Chromosomal disorders	Number of cases (%)
22q11.2 microdeletion	17 (43.6)
Trisomy 21	9 (23.1)
Trisomy 18	1 (2.6)
1q43 deletion	1 (2.6)
Mosaic trisomy 16	1 (2.6)
Partial trisomy of chromosome 22 q11.22 to qter (der(22)(qter -q11.22::p11.1-qter)dn	1(2.6)
Microdeletion of chromosome 10 del(10)(p1?4)	1 (2.6)
Microduplication of chromosome 7 and microdeletion of chromosome 13 (arr[hg19] 7p22.3p21.2(54,215–15,790,648) × 3,13q33.3q34(107,864,778–115,092,619) × 1 mat)	1 (2.6)
Inverted duplication of chromosome 20 (mosaic)(arr 20p13(70,579-,175) × 1,20p13p11.22(1,751,153–21,381,326) × 3)	1 (2.6)
Monogenic disorders	
CHARGE syndrome	2 (5.1)
Cornelia De Lange syndrome	1 (2.6)
Alagille syndrome (*JAG1*)	2 (5.1)
Homozygous *PCDH12* gene, Diencephalic-Mesencephalic Junction syndrome	1 (2.6)
Total	39

### Procedures Before Complete Surgical Repair

A cardiac procedure prior to a complete ToF repair was required in 66/400 (16.5%) of patients (Table 2), of which 174 (44%) were female and 224 (56%) were male. Eleven of the patients had more than one procedure. The median age at the first procedure was 24 days (IQR 7.3 to 69.3 days) and the median weight was 3 kg (IQR 2.76 to 3.86 kg). Fifteen patients (3.8%) underwent a cardiac procedure in the first week of life, and 18 (4.5%) within two weeks of life. A procedure prior to complete ToF repair was more frequently required in those babies in whom a prenatal diagnosis had been made (19.7% vs 12%, *p* = 0.039). Four patients (6%) out of 66 died before complete ToF repair: two after a modified Blalock-Thomas-Taussig (BTT) shunt, one after a cardiac catheter procedure, a right outflow tract stent, and one death was unrelated to any cardiac procedure. Two of these patients were born prematurely and 3/4 had extra-cardiac abnormalities. The temporal distribution of procedures and nature of interventions across the cohort is summarized in Supplementary Table S1.

**Table 2:** Procedures undertaken in 66 patients before complete ToF

Procedure type	Number of procedures
Catheter-based intervention	
PV/RVOT catheter procedure (balloon/stent)	49 (32/17)
PA catheter procedure (balloon/stent)	11 (10/1)
PDA stent	6
Reintervention to shunt (balloon/stent)	3 (1/2)
Surgical Operation	
Modified R/L Blalock-Taussig-Thomas shunt	15
Central shunt	3
Upsizing of shunt	3
RVOT surgical procedure	2
Total	92

*PDA* patent ductus arteriosus, *PA* pulmonary artery, *PV* pulmonary valve, *RVOT* right ventricular outflow tract

### Complete Surgical Repair

Complete repair of ToF was performed in 396/400 (99%) patients at a median age of 6 months (IQR: 4.0–8.0 months) and a median weight of 6.7 kg (IQR 5.8–7.8 kg). Patients with prior right ventricular outflow tract (RVOT) stent, patent ductus arteriosus (PDA) stent, a BTT shunt or no prior cardiac procedures underwent complete ToF repair at a median age of 3.5 months (IQR: 2.3–5.0 months), 4.5 months (IQR: 4.0–6.5 months), 8.0 months (IQR: 7.0–8.8 months), and 7.0 months (IQR: 4.0–8.0 months), respectively. A statistically significant difference in age at complete ToF repair was observed across these subgroups (*p* = 0.001). Pairwise comparisons of age at repair demonstrated statistically significant differences between the RVOT stent and BTT shunt groups (*p* = 0.001), PDA stent and BTT shunt groups (*p* = 0.023), RVOT stent and no prior procedure groups (*p* = 0.003), and BTT shunt and no prior procedure groups (*p* = 0.016). No significant differences were observed between the RVOT stent and PDA stent groups (*p* = 0.353) or the PDA stent and no prior procedure groups (*p* = 0.283). The 30-day postoperative survival rate was 99.5%.

### Procedures After Complete Surgical Repair

Reintervention after complete ToF repair was required in 100/396 (25.3%). Reintervention rate was 3.3% (13/396) at 30 days, 10.6% (42/396) at 1 year, and 19.2% (76/396) at 5 years. During the same admission, 15% of patients (15/100) required an additional procedure following complete ToF repair, whereas 85% (85/100) underwent further intervention at a later stage after discharge. Such procedures were necessary on the right ventricle outflow tract/pulmonary valve, branch pulmonary arteries, mitral valve, or patients needed a pacemaker implantation (Table 3). Thirty-four patients needed more than one procedure.

**Table 3:** Procedures after complete ToF repair within study period of 18 years

Procedure type	Number of procedures
Reintervention	
PA catheter procedure (balloon/stent)	35 (20/15)
PV/RVOT catheter procedure (balloon/stent)	20 (18/2)
Transluminal pulmonary valve replacement	4
Balloon of cardiac conduit	1
ASD transluminal closure	1
Re-operation	
RVOT surgical procedure (reconstruction, muscle resection, patch)	41
PV surgical procedure	19
Pulmonary arterioplasty	16
RV to PA homograft	4
Residual VSD closure	5
MV procedure (regurgitation/supramitral membrane)	4 (3/1)
PPM procedure (mixed)	2
Vascular ring division	2
Total	154

*ASD* atrial septal defect, *MV* mitral valve, *PA* pulmonary artery, *PDA* patent ductus arteriosus, *PPM* permanent pacemaker, *PV* pulmonary valve, *RVOT* right ventricular outflow tract, *RV* right ventricle, *VSD* ventricular septal defect

Four patients required a right ventricle to pulmonary artery conduit within the study period after having had a complete ToF repair with transannular patch. One of the patients had a significant restenosis of the right ventricular outflow tract and left pulmonary artery, which initially was ballooned. The other three patients had significant free pulmonary regurgitation causing right ventricular dysfunction.

### Freedom from Reintervention

**Fig. 1:**
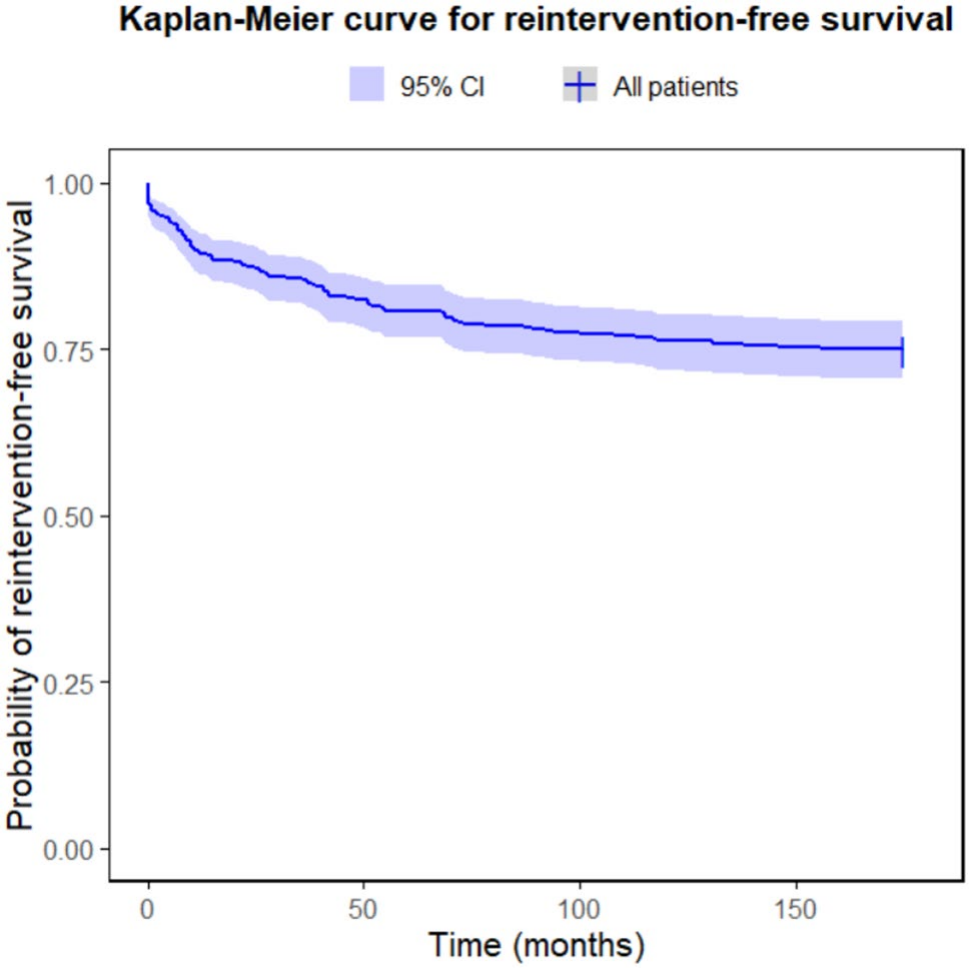
Kaplan–Meier curve for intervention-free survival

After complete surgical repair, freedom from reintervention was calculated using the Kaplan–Meier (Fig. 1) estimate with 95% confidence intervals (CI). Freedom from reintervention was 89.4% (95% CI: 86.4%–92.5%) at 1 year, 87.4% (95% CI: 84.2%–90.7%) at 2 years, and 80.8% (95% CI: 77.0%–84.8%) at 5 years. Among the patients, a small subset—27 out of 396 (6.8%)—required procedures both prior to and following the complete repair of ToF. The presence of a concomitant genetic diagnosis did not significantly alter the likelihood of requiring a palliative procedure before or after complete repair 34.2% vs 34.1%.

### Length of Hospital Stay After Procedures Before Complete Surgical Repair

Excluding the four patients who died prior to complete ToF, the median length of stay (LoS) at the time of the first procedure was 4 days (IQR: 1.0–10.0 days) for those who underwent PV, RVOT, or PA catheter interventions (*n* = 35/62), compared with 8 days (IQR: 6.0–16.0 days) for those who received a BTT shunt procedure (*n* = 18/62). The differences were statistically significant (*p* = 0.0049).

### Post-operative Length of Hospital Stay After Complete Surgical Repair

The overall postoperative median length of stay (LoS) for all patients undergoing complete repair was 6 days (IQR: 4.0–9.0 days). Subgroup analysis showed the highest median LoS following complete ToF repair in patients who had undergone a BTT shunt at 8.0 days (IQR: 7–14). Patients who underwent a previous RVOT stent procedure had a median LoS of 5.4 days (IQR: 3.2–8.0 days), whereas those with a PDA stent had the shortest median LoS at 4.2 days (IQR: 3.7–7.5 days). Although variations in median LoS were observed across the subgroups, the differences were not statistically significant (*p* = 0.068). In the univariable analysis (supplementary Table S2), chromosomal or genetic comorbidities, prematurity, and having undergone a procedure prior to complete ToF repair were associated with prolonged LoS (*p* < 0.1). Age and weight at the time of repair were not significantly associated with LoS. In the multivariable model, chromosomal or genetic comorbidities remained significantly associated with extended LoS (HR: 0.66; 95% CI: 0.46–0.94; *p* = 0.021), as did undergoing a procedure prior to complete ToF repair (HR: 0.7; 95% CI: 0.54–0.93; *p* = 0.012). Prematurity was not statistically significant (HR: 0.81; 95% CI: 0.57–1.16; *p* = 0.248).

### Overall Survival of the Cohort

The median length of follow-up was 8 years (IQR 4–12). There were 7 deaths (1.8%) recorded following ToF repair.

The actuarial survival for the whole group of 410 patients diagnosed with ToF was as follows: 98.5% at one month, 97.8% at 2 months, 96.6% at 6 months, 96.2% at 1 year, 95.5% at 2 years, and 95.2% at 3 years of age.

## Discussion

This study reports outcomes of classic ToF at a single surgical center over an 18-year period. Our cohort confirms good postoperative survival after complete repair of ToF, with mortality < 1% at 30 days and no deaths beyond 2.5 years after birth. This is consistent with the previous reports [7], UK National reported data [6], and a recent meta-analysis of 21,427 case studies [8]. Factors such as staged repairs, non-valve-sparing operations, and genetic abnormalities may influence mortality risks, particularly in the early post-surgical period.

The timing of surgical repair for ToF is a critical factor in the patient outcomes. Current guidelines of The American Association for Thoracic Surgery 2022 Expert Consensus Document [5] suggest that the optimal window for complete surgical correction is between 3 and 6 months of age. This recommendation is based on the potential to reduce the length of hospital stay and the rate of adverse events. Our series indicates a median age of 6 months for complete ToF repair and is consistent with the recommended timeframe. It is acknowledged that complete repair before 3 months or beyond 12 months of age may be less favorable [5, 7–11], increasing the hospital stay and number of reinterventions.

Our study provides data on the LoS associated with various cardiac procedures that these patients may undergo. It highlights that the median LoS for complete ToF repair is typically a week. Patients treated initially with a BTT shunt may have a slightly longer hospitalization after complete ToF repair, compared to those who underwent RVOT or PDA stent procedures, who typically have a shorter median hospital stay. However, these differences were not statistically significant. There was an anticipated difference between patients with initial RVOT or PDA stents proceeding to a complete ToF repair sooner than those with BTT shunts; the median ages were 3.5 months, 4.5 months, and 8 months, respectively. This reflects our institutional practice to avoid BTT and preference for a catheter-based procedure in premature babies or those with low birth weight, in whom decision-making is tailored to the anatomical substrate and other patient factors. Thus, it is anticipated that an earlier repair or reintervention is required.

In addition, our analysis indicates that chromosomal or genetic comorbidities and undergoing a procedure prior to complete ToF repair are associated with prolonged hospital stay. These findings underscore the importance of early identification and strategic planning in patients with syndromic diagnoses or those likely to require staged interventions. Although prematurity initially appeared linked to increased length of stay, it did not retain significance in the multivariable model, potentially due to confounding clinical factors or consistent perioperative management across groups. Notably, age and weight at the time of repair were not associated with hospital stay duration, suggesting that timing alone may be less influential than the patient’s baseline characteristics and procedural history.

Although this study did not specifically focus on the antenatal group, there was a statistically significant difference in the number of patients who underwent at least one procedure before complete ToF repair between those with antenatal and postnatal diagnoses. Patients with antenatal diagnoses required such procedures more frequently (19.7%) compared to those with postnatal diagnoses (12%).This might be a reflection of the more severe forms of ToF (with hypoplastic pulmonary artery) being detected during routine prenatal screening [12]. The data we present can assist healthcare professionals in antenatal discussions with parents.

The data analysis revealing that 16.5% of patients required cardiac procedures prior to complete ToF repair aligns with findings from Mimic et al.’s 2013 report [13]. Notably, some patients underwent multiple procedures with the first in the neonatal period (median age 24 days; IQR 7–72 days). This information is crucial for medical professionals counseling the parents and planning the treatment timeline for patients with ToF.

Over time, there has been a gradual shift away from traditional surgical approaches of modified BTT shunts toward catheter-based strategies, including RVOT stenting and arterial duct stenting, resulting in shorter hospital stays. This transition reflects broader trends observed in other centers [14] and is likely driven by advancements in interventional techniques and equipment over time. Emerging evidence suggests that these catheter-based approaches may be associated with improved pulmonary artery development compared to conventional shunting, providing a compelling rationale for their increased adoption.

The systematic review by Romeo et al. [8] in 2020 highlights the fact that reinterventions and reoperations are not uncommon following complete repair of ToF, with pooled data indicating early reintervention rates of 2.3% and reoperation rates of 1.7%.

The 2022 annual report of the German Registry for Cardiac Operations and Interventions in Congenital Heart Disease [15] revealed that 21% of patients underwent repeat interventional or surgical procedures following complete ToF repair. Our study similarly found that a significant proportion (25.3%) required further reinterventions (operations or catheter interventions). Following corrective ToF repair, the rate of reinterventions gradually increased over time: 3.3% of patients required additional procedures within 30 days of the repair, 10.6% within the first year, and 19.2% by 5 years. These follow-up procedures were predominantly focused on the right ventricular outflow tract/pulmonary valve, branch pulmonary arteries, and the mitral valve, while some patients required implantation of a pacemaker. The commonest reintervention was RVOT and/or pulmonary artery balloon or stent (*n* = 55). It is also noted that a subset of these patients underwent multiple reinterventions.

Our study underscores the importance of systematic follow-up and management strategies [16] for patients undergoing ToF repair to ensure optimal outcomes and address any complications that may arise after corrective surgery.

This study found that while reinterventions after complete ToF repair were common, freedom from reintervention was 89.4% at 1 year, 87.4% at 2 years, and 80.8% at 5 years. This is comparable to other published data [13, 17] and suggests that the majority of patients do not require additional surgical intervention within the first five years following ToF repair, highlighting the effectiveness of the procedure in the medium term. However, it is important to note that the risk of reintervention may be influenced by various factors, including the specifics of the surgical procedure, the patient’s condition, and the criteria used for determining the need for reintervention.

Four patients required a right ventricle to pulmonary artery homograft during childhood after having had a complete ToF repair with a transannular patch. The necessity for a conduit is a standard procedure in cases with pulmonary atresia [18], but it has been reported [13] also in reoperations of classic ToF. Hence, it is crucial to communicate this information to parents, even though it is applicable to only 1% of the patients in our study of 396 patients.

Our study highlights important aspects of managing ToF. It is noted that a small subset of patients, 6.8%, required procedures both before and after the complete repair of ToF. This emphasizes the importance of individualized treatment strategies and thorough preoperative counseling to set realistic expectations for outcomes and quality of life.

An additional and progressive mitral valve abnormality needing a surgical intervention was found in four patients, one with supramitral membrane and three with severe regurgitation. This is an uncommon [19] but important finding that may influence the management approach for patients. The presence of a supramitral membrane was first reported by A. Hohn in 1968 [20] in a patient with unoperated ToF who died due to respiratory disease. In cases where such abnormality is present at early infancy, the decision between catheter-based or shunt procedure and complete surgical intervention requires careful consideration.

Tetralogy of Fallot has been linked to genetic abnormalities, with postnatal studies indicating a notable incidence (14 to 30%) [7, 9, 13, 20, 21] of 22q11.2 microdeletion (5.8 to 15%) [7, 9, 21, 22], with the higher number referring to the combined classic and ToF with pulmonary atresia group of patients. Moreover, with the introduction of techniques such as whole exome or whole genome sequencing, the diagnostic yield is likely to increase still further [23]. In our series, 13% (5/38) of patients with genetic abnormalities had monogenic disorders, which would not be diagnosed with the conventional comparative genomic hybridization array method. The lower prevalence of genetic conditions in our series (9.59%), including a 4.3% occurrence of 22q11.2 microdeletion, might reflect ascertainment bias as we only included liveborn cases, and the finding of a genetic condition may impact parental decision-making to continue with pregnancy or not. Furthermore, the incidence of monogenic disorders might also be underestimated as this type of testing has only been used selectively in the last eight years. Our data show that the presence of a concomitant genetic diagnosis did not significantly alter the likelihood of the requirement of a palliative procedure before or after complete repair of ToF (34.2% vs 34.1%). A similar observation has been reported by Blais et al. in 2021 [7].

## Limitations

A comprehensive search of our database was undertaken, but there might be cases which were not included due to death prior to intervention. However, from our fetal cardiac database, this number is likely to be low. It is acknowledged that decision-making concerning the management of TOF has progressed over time, with a transition toward earlier corrective surgery instead of palliation. This shift aligns with advancements in CHD management, which have also influenced approaches to pulmonary valve replacement throughout the study period.

## Conclusion

One in six patients born with tetralogy of Fallot required early intervention to augment pulmonary blood flow. With current surgical practices, ToF can be repaired with a low procedural mortality rate and promising medium-term outcomes and survival into early adulthood. In those requiring early augmentation of pulmonary blood flow, shorter hospital stay was noted for patients who underwent RVOT or ductal stent compared to those that underwent BTT shunt. An earlier complete repair was required in those who underwent interventional rather than surgical procedures. Despite the high success rates of initial surgeries, the likelihood of requiring reinterventions remains high throughout a patient’s life. This communication is essential for informed consent, particularly when a prenatal diagnosis is made so that expectant parents have realistic expectations for the surgical outcome and potential future interventions.

## Supplementary Information

Below is the link to the electronic supplementary material.

Supplementary file1 (DOCX 21 KB)

Supplementary file2 (DOCX 17 KB)

## Data Availability

No datasets were generated or analysed during the current study.
