# Supplementary material for: Outcome of Tetralogy of Fallot Through Initial Palliation and Surgical Repair
Source: Pediatr Cardiol. 2025 Sep 24;47(5):2250–7. doi: 10.1007/s00246-025-04021-1 (PMC13144264; doi:10.1007/s00246-025-04021-1)
Supplement: Supplementary file 1 — Supplementary file1 (DOCX 21 KB) [file 246_2025_4021_MOESM1_ESM.docx]

**Supplementary Table S1.** Distribution of procedures and nature of interventions before compete ToF repair across the 18 year cohort.

BTT- Blalock- Thomas -Taussig, PA- pulmonary artery, PDA – patent ductus arteriosus , PV- pulmonary valve, RVOT-right ventricular outflow tract
