# Supplementary material for: Outcome of Tetralogy of Fallot Through Initial Palliation and Surgical Repair
Source: Pediatr Cardiol. 2025 Sep 24;47(5):2250–7. doi: 10.1007/s00246-025-04021-1 (PMC13144264; doi:10.1007/s00246-025-04021-1)
Supplement: Supplementary file 2 — Supplementary file2 (DOCX 17 KB) [file 246_2025_4021_MOESM2_ESM.docx]

**Supplementary Table S2.**

Cox proportional hazards models were used to evaluate the association between selected clinical predictors and postoperative length of stay (LoS) following complete ToF repair. Univariable models were first applied to assess the effect of each variable, including age at repair (months), weight at repair (kg), presence of chromosomal or genetic comorbidities, prematurity and having undergone a procedure prior to complete ToF repair. In the univariable analysis, the presence of chromosomal or genetic comorbidities, prematurity and undergoing a procedure prior to complete ToF repair were associated with a longer LoS (p < 0.1). Age at repair and weight at repair were not significantly associated with LoS.

Variables with a univariable *p*-value < 0.1 were included in the multivariable Cox proportional hazards model. After adjusting for potential confounders, the presence of chromosomal or genetic comorbidities significantly associated with prolonged LOS (HR: 0.66; 95% CI: 0.46–0.94; *p* = 0.021). Undergoing a procedure prior to complete ToF repair was also associated with longer LoS (HR: 0.70; 95% CI: 0.54–0.93; *p* = 0.012). Prematurity was not statistically significant in the multivariable model (HR: 0.81; 95% CI: 0.57–1.16; *p* = 0.248).



| **Multivariable cox proportional hazards model** | | |  |  |  |  |  |  |
| --- | --- | --- | --- | --- | --- | --- | --- | --- |
| **Predictor** |  | **Univariable model** | |  |  | **Multivariable model** | |  |
|  |  | HR | 95% CI | P value |  | HR | 95%CI | P value |
| Age at repair (months) |  | 1.000 | 0.9934 - 1.009 | 0.713 |  |  |  |  |
| Weight at repair (kg) |  | 1.010 | 0.984  - 1.039 | 0.417 |  |  |  |  |
| Genetic or chromosomal comorbidities | | 0.599 | 0.421 - 0.851 | 0.004 |  | 0.655 | 0.458 - 0.937 | 0.021 |
| Prematurity |  | 0.733 | 0.517 - 1.037 | 0.079 |  | 0.812 | 0.570 - 1.156 | 0.248 |
| Pre ToF repair procedure |  | 0.672 | 0.512 - 0.883 | 0.004 |  | 0.703 | 0.535 - 0.925 | 0.012 |
| HR, Hazard ratio; CI, Confidence interval | |  |  |  |  |  |  |  |
